# Response to Comments on Whiley *Legionella* Risk Management and Control in Potable Water Systems: Argument for the Abolishment of Routine Testing. *Int. J. Environ. Res. Public Health* 2017, *14*, 12

**DOI:** 10.3390/ijerph14010103

**Published:** 2017-01-21

**Authors:** Harriet Whiley

**Affiliations:** Health and the Environment, School of the Environment, Flinders University, GPO Box 2100, Adelaide, South Australia 5001, Australia; Harriet.Whiley@flinders.edu.au; Tel.: +61-8-7221-8580

I would like to thank Collins and Walker for their considered comments and for acknowledging that this is an area urgently requiring more research to improve *Legionella* control and management strategies [1].

I agree with Collins and Walker’s conclusion that the optimum solution would be a water system management strategy that, using an improved *Legionella* detection method, combines risk assessment, control measures, and routine testing. However, this is currently not an option, as a *Legionella* detection method that adequately represents the public health risk has not yet been identified [2].

I also suggest that the ‘zero-tolerance’ approach to *Legionella* utilized in many UK hospitals is an example of false confidence in the culture detection results. It demonstrates how a negative detection result may cause managers to assume their system is ‘*Legionella*-free’. However, as discussed in the commentary, this is not guaranteed [2].
